# Interplay between Nrf2 and ROS in regulating epithelial-mesenchymal transition: implications for cancer metastasis and therapy

**DOI:** 10.1007/s11033-025-10731-9

**Published:** 2025-06-23

**Authors:** Yi Xu, Shuning Hu, Rui Chen, Sheng Xu, Guangyang Yu, Lili Ji

**Affiliations:** 1 Department of Pathology, Key Laboratory of Microenvironment and Translational Cancer Research, Medical School of Nantong University, Nantong, Jiangsu 226001 China; 2 Department of Pathology, Lianyungang Affiliated Hostital Of Nanjing University of Chinese Medicine, Lianyungang, Jiangsu 222004 China

**Keywords:** Epithelial-mesenchymal transition (EMT), Nrf2, ROS, Cancer stem cells, Oxidative stress

## Abstract

Epithelial-mesenchymal transition (EMT), including developmental (Type I), wound healing (Type II), and pathological (Type III) subtypes, constitutes a critical driver of cancer metastasis. This review analyzes the redox interplay between nuclear factor erythroid 2-related factor 2 (Nrf2) and reactive oxygen species (ROS) in EMT regulation and cancer progression. Nrf2 maintains redox homeostasis through antioxidant gene activation while paradoxically promoting tumor survival and drug resistance via Keap1-dependent degradation and phosphorylation-mediated stabilization. ROS generated through mitochondrial and NADPH oxidase pathways exhibit dual functionality: moderate levels activate EMT transcription factors to drive metastasis and cancer stem cells (CSCs) plasticity, whereas excessive ROS induce apoptosis and ferroptosis. While Nrf2 typically suppresses EMT through ROS neutralization and epithelial integrity preservation, chronic Nrf2 activation in CSCs paradoxically sustains metastatic potential through redox buffering. This synthesis delineates the spatiotemporal regulation of Nrf2-ROS-EMT networks across tumor microenvironments, emphasizing therapeutic opportunities through redox balance modulation and pathway-specific Nrf2 inhibition in advanced malignancies.

## Introduction

WHO’s 2023 online cancer statistics report 19.29 million new cases and 9.96 million deaths globally, with a mortality rate of 52% [1]. According to the 2023 data published online by the Chinese Center for Disease Control and Prevention, China’s national as well as local cancer burdens from 2005 to 2020 were evaluated [2]. The study estimated that the burden of cancer-related mortality in China in 2020 was 2.397800, an increase of 21.6% over 2005, and the burden of premature mortality was 56.599 million person-years, an increase of 5.0%. Most cancer-related mortality arises from metastatic progression rather than primary tumor burden. The infiltration and metastasis of malignant tumors are critical factors contributing to their poor prognosis. Metastasis involves a series of processes wherein primary tumor cells gradually gain invasive capabilities, colonize distant organs, and proliferate through blood, lymphatic vessels, or by directly infiltrating adjacent structures. Epithelial-mesenchymal transition (EMT) plays a pivotal role in the process of tumor invasion and metastasis [3]. Therefore, elucidating the molecular mechanisms governing EMT in tumor invasion and metastasis is critical for improving cancer treatment outcomes.

Discovered in 1994 [4], Nuclear Factor Erythroid 2-related Factor 2 (Nrf2) is a critical regulator of antioxidative stress, essential for the antioxidant defense system and cellular protection against oxidative stress. Nrf2 functions as an antioxidant factor, regulating intracellular reactive oxygen species (ROS)levels. The increase in intracellular ROS levels holds great significance in biological systems. ROS exhibit dual functionality: physiological levels sustain homeostatic signaling, whereas pathological accumulation induces oxidative damage. Oxidative stress contributes substantially to the pathogenesis of various diseases, particularly malignancies. The ability of ROS to promote phenotypic plasticity during tumor formation and confer stem-like properties to cancer cells has become a broad field of research and is considered a potential factor in EMT. High levels of ROS can facilitate the EMT process through the activation of multiple transcription factors.

This paper discusses the cellular biological mechanism of tumor metastasis and the effect of the Nrf2-ROS pathway on tumor EMT progression.

## Key concepts and EMT classification

### Definition of EMT

First characterized in 1960, EMT is an evolutionarily conserved reprogramming process essential for embryonic development, wound healing, and pathological conditions including fibrosis and cancer metastasis [5]. This reversible phenotypic switch involves coordinated molecular remodeling of epithelial cells characterized by apical-basal polarity loss, cytoskeletal remodeling, and cell adhesion complex disassembly. These alterations collectively confer mesenchymal properties, enhancing cellular motility and invasive capacity [6]. EMT facilitates epithelial cell detachment from primary sites and subsequent migration through extracellular matrix remodeling. In malignancy, EMT drives metastatic dissemination via phenotypic conversion of epithelial cells to mesenchymal-like states [7]. This process critically contributes to tumor progression and metastatic cascade. Thus, studying the molecular mechanisms of EMT and targeting these pathways may significantly enhance clinical outcomes by improving both overall survival and patient quality of life in oncology.

**Fig. 1:**
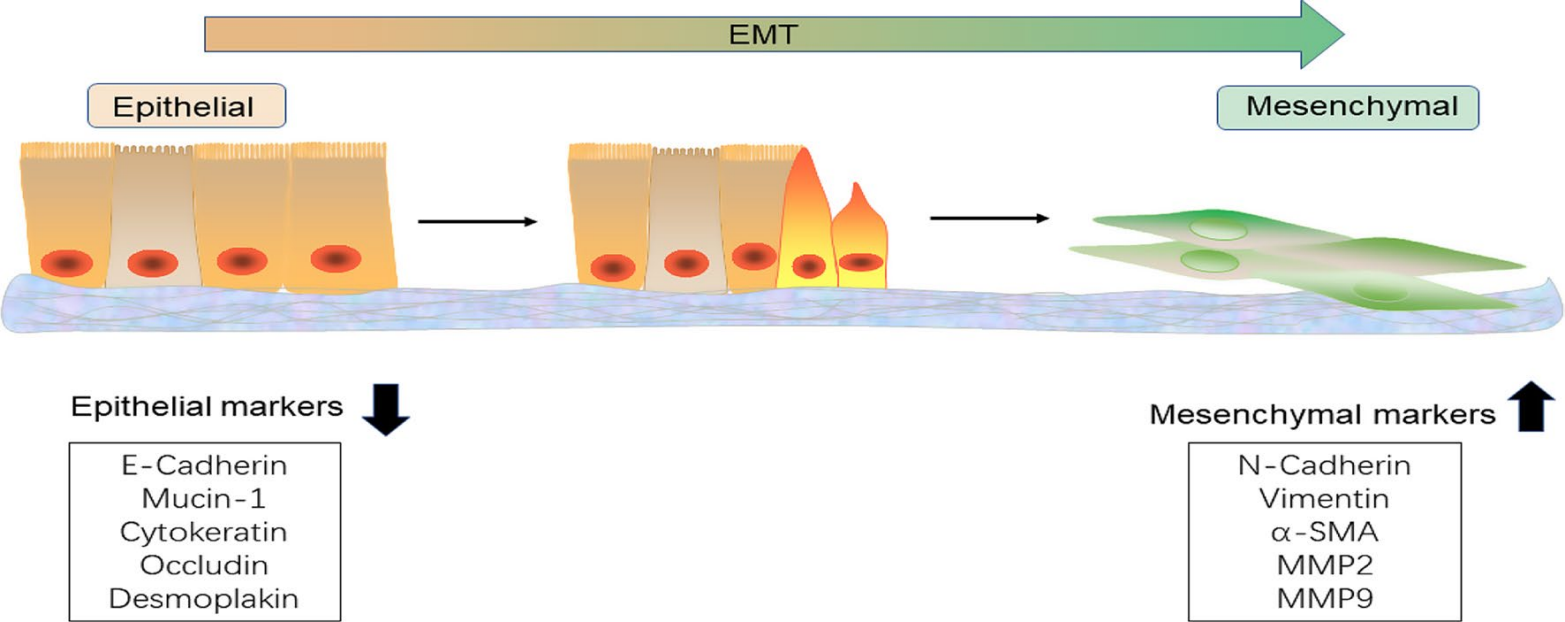
EMT and Key Biomarkers (Created with PowerPoint). This schematic depicts EMT, highlighting morphological and molecular shifts. Epithelial cells (left) exhibit cuboidal shape, E-cadherin-based junctions, and epithelial markers (E-Cadherin, Mucin-1, CK19/18/8, Occuluding and Desmoplakin). Mesenchymal cells (right) adopt a spindle-like structure with upregulated mesenchymal markers (N-cadherin, Vimentin, α-SMA, and MMP2/9)

Morphologically, EMT induces spindle-shaped cytoskeletal reorganization, with molecular progression monitored through characteristic biomarkers. Key epithelial markers (e.g., E-cadherin) are suppressed, whereas mesenchymal markers (e.g., N-cadherin, vimentin) are induced [8] (Fig. 1). This molecular signature enables tumor cell dissemination from primary lesions, correlating with aggressive progression and poor clinical outcomes [9].

### EMT classification

Three EMT subtypes are defined by biological context. First, type I EMT affects embryo implantation and development. Mesenchymal derivatives undergo mesenchymal-epithelial transition (MET) to regenerate epithelial tissues during embryogenesis.

Secondly, Type II EMT is related to tissue repair following injury, regenerative mechanisms, and fibrotic tissue remodeling in various organs. It is is triggered by immune cell cytokines (macrophages, neutrophils) responding to antigenic stimuli. Fibroblast activation and myofibroblast differentiation are driven by TGF-β, Notch, and Wnt pathways, generating disorganized extracellular matrix components. Persistent matrix deposition culminates in irreversible fibrotic remodeling.

Type III EMT facilitates tumor metastasis. The metastasis of cancer is a gradual process, with EMT being the first step. EMT is initiated by losing cellular polarity and disrupting cellular adhesions, including desmosomes, tigh/adherens/gap junctions. This transition enhances cells’ migratory and infiltrative abilities. Core regulatory pathways include TGF-β, Wnt, Notch, and growth factor receptor systems. EMT reprograms ~ 400 genes, including core transcription factors (SNAIL, TWIST, ZEB) that suppress epithelial markers like E-cadherin [10]. Besides metastasis, by increasing resistance to chemo-, radio-, and immunotherapies, EMT also contributes to primary tumor progression.

## Nrf2 structure, regulation and function

### Nrf2 structure

Nrf2 is a regulatory protein involved in the process of oxidation. This was first observed in 1994 [4]. Functioning as a critical transcriptional regulator, Nrf2 undergoes nuclear translocation in response to increased ROS accumulation or oxidative stress conditions. This nuclear translocation enables its interaction with antioxidant response elements (AREs) located within the regulatory regions of target genes, subsequently activating their transcriptional programs to mediate cellular antioxidant responses. These genes include intracellular antioxidant enzymes, phase II enzymes that facilitate detoxification, and additional antioxidant enzymes.

**Fig. 2:**
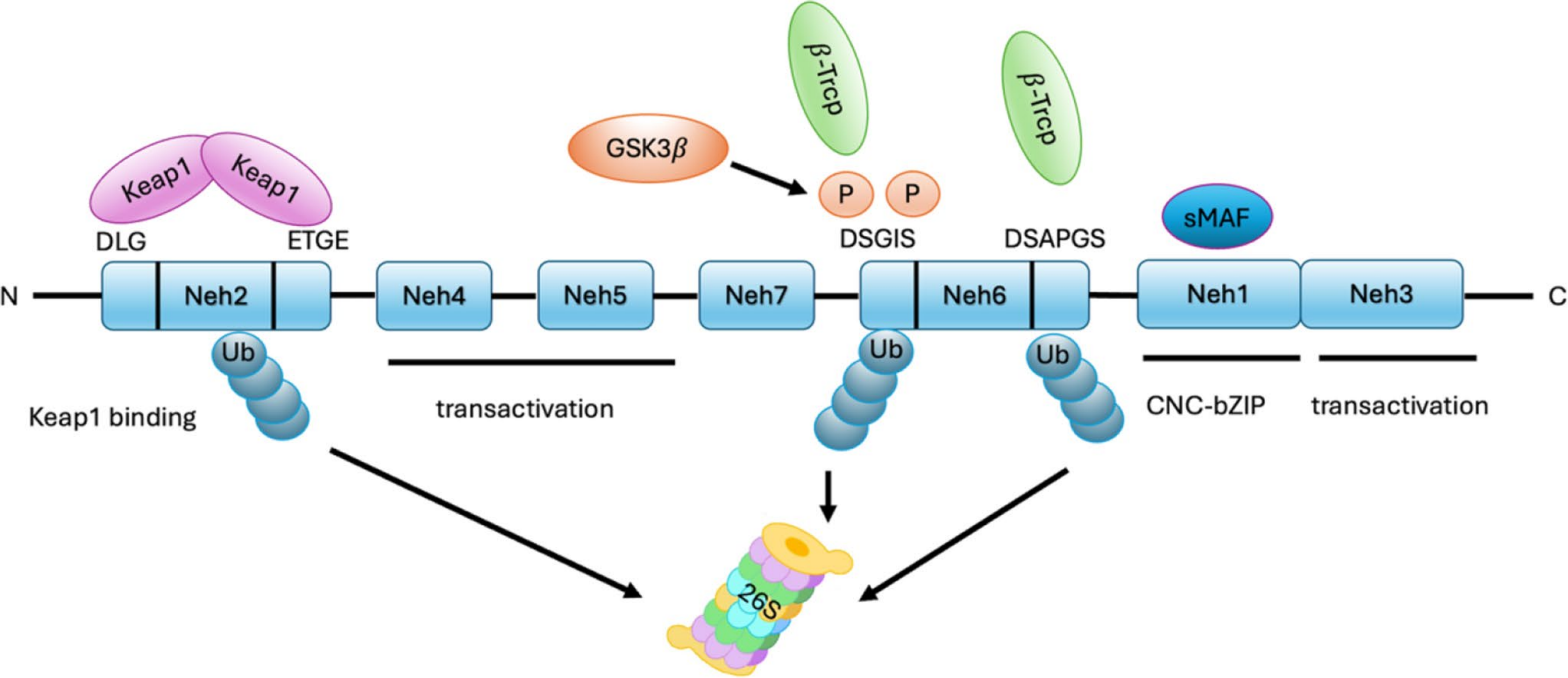
Domain Architecture and Functional Motifs of Nrf2 (Created with PowerPoint). This schematic illustrates the multi-domain structure of Nrf2, a master regulator of cellular defense against oxidative stress. The protein comprises seven conserved Neh domains (Neh1-Neh7), each contributing distinct functional roles. This architecture highlights Nrf2’s dual regulation (Keap1/β-TrCP) and its role in orchestrating antioxidant gene networks

It is evident that Nrf2, an acronym for the gene NFE2L2, is a transcription factor characterized by a highly conserved basic leucine zipper (BZIP) domain. This structure is also present in the Cap-n-collar (Cnc) family, comprising nuclear factor-erythroid 2 (NFE2), Nrf1, Nrf2, and Nrf3 [11]. Among these, Nrf2 is the most well-known family member due to its cytoprotective role in oxidative stress responses [12]. By preserving the redox homeostasis of cell, Nrf2 modulates the adaptive responses to exogens and oxidative stress. Nrf2 exhibits a multi-domain architecture consisting of seven conserved functional regions, designated Neh 1 through Neh 7 [13]. The Neh1 encompasses the characteristic CNC-bZIP motif, mediating both Maf protein binding and DNA recognition. The N-terminal Neh2 domain hasDLG and ETGE sites which recruitKeap1, a regulator that functions in a negative manner with regard to Nrf2. It is evident that the C-terminal Neh3 domain, through the recruitment of helicase DNA-binding protein and CHD ATPase, participates in upstream gene transcription based on the cullin E3 ubiquitin ligase (Cul3) complex. The Neh4 and Neh5 domains function as transcriptional coactivator binding sites, interacting with steroid receptor SRC3 and cAMP-response element-binding protein (CREB) to bind with target gene regulation. The Neh6 domain conserved DSGIS and DSAPGS motifs that mediate binding to beta-transducin repeat-containing proteins (β-TrCP). This interaction facilitates Nrf2 degradation through Roc1-dependent ubiquitination, representing a redox-independent regulatory mechanism for Nrf2 activity control. The final domain, Neh7, can inhibit transcription by binding to the retinoic X receptor [14] (Fig. 2).

### Nrf2 regulation

The complex mechanisms governing Nrf2 activation, and its target gene expression are currently under intensive investigation.

#### Keap1-Mediated degradation mechanism of Nrf2

Keap1 functions as a molecular regulator that detects cellular oxidative stress and negatively modulates the intracellular protein levels of Nrf2. Through a Cullin3-dependent E3 ubiquitin ligase, Keap1 captures Nrf2 and rapidly ubiquitinates it, ultimately targeting it for proteolytic processing via the 26 S proteasome. This process maintains low levels of Nrf2 within the cell under unstimulated conditions. However, in conditions of oxidative stress, structural modifications in Keap1 enable the liberation of Nrf2 from its inhibitory complex. Subsequently, Nrf2 undergoes nuclear translocation, where it associates with Maf proteins to form a heterodimeric complex. This Nrf2-Maf heterodimer can recognize the antioxidant response element (ARE) gene sequence as well as induce expressed genes associated with antioxidant and metabolic processes [15].

#### Phosphorylation of Nrf2 and its degradation mechanism

Nrf2 is downstream target of several protein kinases. These include protein kinase C (PKC), casein kinase 2 (CK2), Fyn tyrosine kinase, and glycogen synthase kinase-3 (GSK-3) [16].Experimental evidence indicates that Nrf2 activity is modulated not only through oxidative stress responses and Keap1-dependent ubiquitination but also via phosphorylation-mediated negative regulation by multiple kinase pathways [17]. For instance, PKC can mediate the phosphorylation of Nrf2. This phosphorylation-dependent mechanism facilitates the release of Nrf2 from the inhibitory complex formed Keap1, enabling nuclear translocation and initiate the activation of downstream gene expression programs. Additionally, the casein kinase 2 (CK2)-mediated phosphorylation of Nrf2 represents a key regulatory step controlling its nuclear import and transcriptional function in neuroblastoma cells [18]. Upon phosphorylation, Fyn translocates to the nucleus and catalyzes the phosphorylation of Nrf2 at tyrosine 568, initiating a cascade of events including nuclear export, ubiquitin-dependent proteolysis, and causing downregulation of Nrf2 target genes [19]. The Neh6 domain of Nrf2 harbors two distinct beta-TrCP recognition motifs, with one of these motifs is regulated by GSK3β activity, leading to its phosphorylation and Keap1-independent ubiquitination and degradation [20]. Another mechanism involves the activation of GSK3β, resulting in the phosphorylation and translocation of nuclear Fyn kinase. The nuclear-localized Fyn then phosphorylates Nrf2 at Tyr568, stimulating its subsequent degradation by the proteasome and export from the nucleus [21].

### Nrf2 function

In tumor initiation and development, Nrf2 plays roles in both tumor suppression and tumor promotion.

#### Antioxidant and cytoprotective roles of Nrf2

As an important antioxidant transcription factor, Nrf2 can reduce ROS levels by activating a series of target genes containing ARE sequences, thus maintaining the relative homeostasis of ROS in cells. These genes are responsible for regulating GSH metabolism, including glutamate-cysteine ligase (GCLC/GCLM), xCT (the glutamate/cystine antiporter), thioredoxin (TXN), and glutathione synthetase (GSS). Another set of genes is responsible for the enzymatic antioxidant system, including NADPH quinone oxidoreductase 1 (NQO1) and selenocysteine-containing antioxidant protein GPX [22]. Therefore, Nrf2 is generally considered to be a key regulator of the cell’s antioxidant reaction. The activation of Nrf2 has been widely established to exert cytoprotective functions in various oxidative stress-related diseases [12]. Consequently, the ability of various natural sources to induce Nrf2 activation and their potential antioxidative and chemopreventive properties were investigated [23]. In healthy tissue, transient activation of the Nrf2 signaling cascade exerts chemopreventive effects through the regulation of diverse gene regulatory networks. During oxidative stress, over 1000 gene promoters with ARE can be activated by Nrf2 [24].

#### Nrf2-Mediated regulation of proliferation genes: basal and inducible expression

Nrf2 can promote tumor cell proliferation by regulating genes such as *PDGFC*, *NOTCH1*, and *VEGFC*, etc. Moreover, Nrf2 facilitates tumor progression by modulating ATF4-mediated transcriptional control of serine/glycine metabolic pathways, thereby accelerating protein biosynthesis in malignant cells [25].

#### The role of Nrf2 in enhancing Anti-Apoptosis and drug resistance in tumor cells

**Fig. 3:**
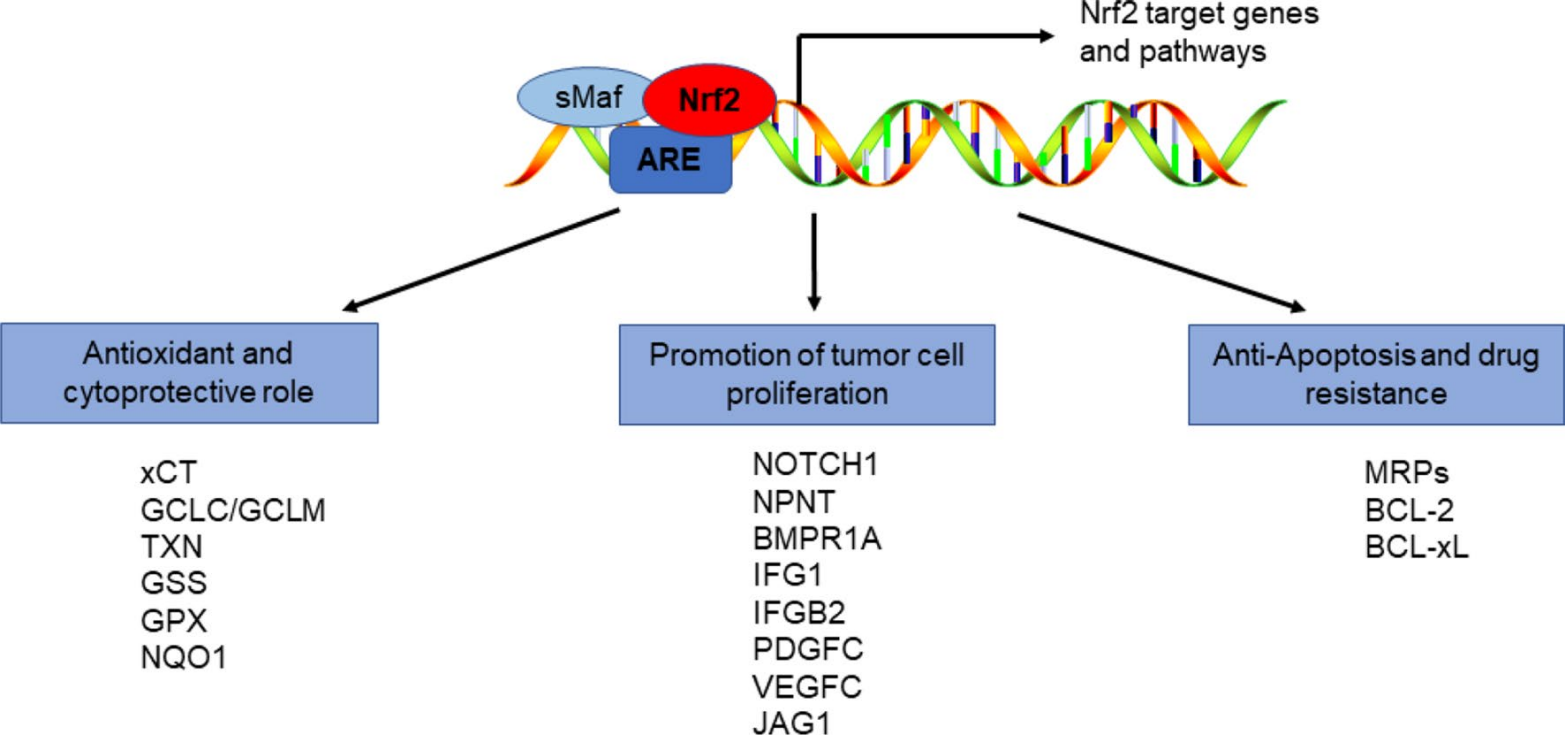
Function of the Nrf2 protein (Created with PowerPoint). This schematic illustrates the dual roles of Nrf2 in cancer biology. Key elements include Nrf2-mediated antioxidant/cytoprotective effects via ARE-driven genes (e.g., GCLC, NQO1) and its tumor-promoting functions through proliferation (NOTCH1, PDGFC), anti-apoptosis (BCL-2, BCL-xL), and drug resistance (MRP transporters, DNA repair pathways). The figure highlights Nrf2’s paradoxical impact: while transient activation protects normal cells, its hyperactivation in tumors drives chemoresistance and progression, underscoring its therapeutic relevance as a target for sensitizing cancer cells to treatment

Whereas transient Nrf2 activation serves as a cytoprotective mechanism in normal cells, constitutive activation in malignancies may facilitate their survival and adaptation to oxidative stress. This could be harmful because of its association with cancer progression and chemoresistance. Nrf2 serves as a transcriptional regulator for several members of the multidrug resistance-associated protein (MRP) family, including MRP1, MRP2, MRP3, and MRP4, which play key roles in cellular defense. Hence, a major factor in tumour resistance to radiotherapy and chemotherapy is likely to be the overactivation of the Nrf2 pathway. Furthermore, Nrf2 can directly block apoptosis through upregulating BCL-2 and BCL-xLexpression, thereby modulating mitochondrial cytochrome c efflux and the activation of caspases-3 and − 7 in response to treatments such as cisplatine and etoposide, which are frequently utilized in antineoplastic therapy [26]. Additionally, studies have demonstrated that Nrf2 can promote restoring radiation-mediated DNA lesions through ROS-independent homologous recombination repair pathways, leading to tumor cell resistance to radiotherapy and chemotherapy [27]. Thus, inhibiting Nrf2 activity may represent a promising therapeutic strategy for increasing the sensitivity of tumor cells to chemotherapy (Fig. 3).

### Nrf2 activation across Cancer types

The Nrf2 signaling pathway demonstrates a high prevalence of activation across various cancer types (Table 1), where it orchestrates tumorigenesis and progression through multifaceted mechanisms including but not limited to antioxidant defense regulation, metabolic reprogramming, and chemotherapy resistance development.

**Table 1:** Summary of Nrf2 pathway activation frequency across cancer types

Cancer Types	Activation Frequency /Positive Rate	Sample Size	Detection Methods
NSCLC	50% (cell lines)	12 cell lines	KEAP1 gene sequencing, LOH analysis (19p13.2 loss of heterozygosity) [28]
	19% (tumor samples)	54 tumor samples	
LUSC	31%	TCGA database	Whole-exome sequencing, RNA sequencing, mutation analysis [29, 30]
LUAD	25%	TCGA database	Whole-exome sequencing, RNA sequencing, mutation analysis [29, 30]
CRC	62.5%	76 tissue samples	Immunohistochemistry (IHC), Real-time quantitative PCR (RT-qPCR) [31, 32]
OSCC	46% high expression of p-Nrf2	110 clinical samples	IHC, qRT-PCR, Western blot [33]
ESCC	55-62.5%	201 samples	IHC, Metabolite assay (mass spectrometry), Gene set enrichment analysis (GSEA) [34]
HNSCC	28%	30 tumor samples	Targeted proteomics (IS-PRM), NGS, Immunofluorescence [35]
Bladder cancer	R34 mutation enriched	TCGA database	Whole-exome sequencing, Mutation analysis (NRF2 R34 site mutations) [36]
HCC	High mutation frequency	TCGA database	KEAP1/NRF2/CUL3 mutation analysis, RNA sequencing [36]
Renal cell carcinoma	28%	50 metastatic sample	Targeted NGS (Nrf2/KEAP1/VHL/FH genes), IHC [37]
THCA	High expression	34 tumor samples	HC, Western blot, qRT-PCR [38]

Key abbreviations: NSCLC (Non-small cell lung cancer); LUSC (Lung squamous cell carcinoma); CRC (Colorectal cancer); OSCC (Oral squamous cell carcinoma); ESCC (Esophageal squamous cell carcinoma); HNSCC (Head and neck squamous cell carcinoma); HCC (Hepatocellular carcinoma); THCA (Thyroid cancer); LOH (Loss of heterozygosity); TCGA (The Cancer Genome Atlas); IHC (Immunohistochemistry); RT-qPCR (Real-time quantitative PCR); NGS (Next-generation sequencing);; IS-PRM (Immunoprecipitation-coupled selected reaction monitoring)

## ROS generation and its function in normal and tumor cells

### Generation of ROS

ROS comprise a group of reactive small molecules derived from oxygen metabolism, encompassing free radical species such as singlet oxygen (^1^O_2_), superoxide anion (O_2_^·−^), and hydroxyl radical (·OH), as well as non-radical oxidants including hydrogen peroxide (H_2_O_2_), lipid peroxides, protein peroxides, and nucleic acid peroxides. The primary endogenous sources of ROS generation are mitochondrial electron transport and the NADPH oxidase (NOX) enzyme family.

### The role of ROS in normal cells

**Fig. 4:**
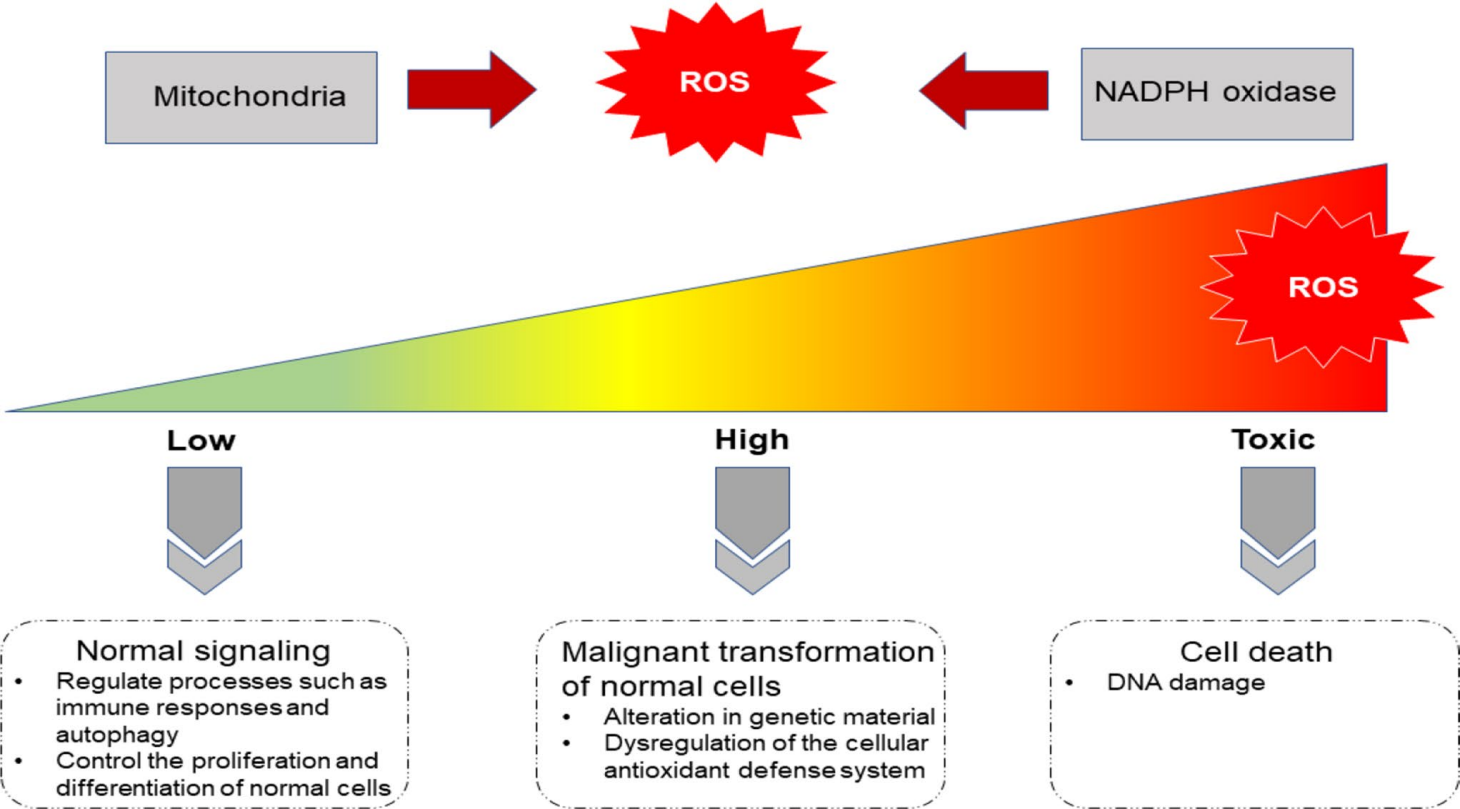
Role of ROS in normal cells (Created with PowerPoint). This figure illustrates the dual role of ROS as redox messengers in cellular regulation. Moderate levels regulate proliferation, differentiation, and autophagy (left), while excess ROS (right) cause oxidative damage, genomic instability, oncogenesis and even cell death. Balanced ROS homeostasis is vital for cell survival; dysregulation drives pathological transformation

Appropriate ROS concentrations are critical for normal cellular function. Research demonstrates that ROS can regulate processes such as immune responses and autophagy by acting as “Redox messengers” [39]. Moderate ROS levels serve as key modulators of proliferation and differentiation in healthy cells, whereas excessive ROS can damage lipids, DNA, and proteins. Consequently, stringent regulation of physiological ROS concentrations is essential for determining the “fate” of cells. The maintenance of physiological ROS homeostasis is vital for normal cell survival; conversely, abnormal ROS accumulation can induce pathological changes (including genetic alterations), driving malignant transformation. Furthermore, excessive ROS accumulation disrupts cellular antioxidant defense homeostasis, a condition strongly implicated in the pathogenesis of various diseases, including cancer [40]. At cytotoxic concentrations, ROS are highly detrimental, including oxidative stress that leads to cellular damage or death (Fig. 4).

### The dual function of ROS in Cancer cells

ROS levels exert dual regulatory effects on neoplastic cell growth and death during distinct stages of carcinogenesis. Elevated ROS can promote tumor initiation and progression by activating oncogenic signaling pathways, inhibiting the function of tumor-suppressor genes, and inducing mutagenesis. Conversely, excessive ROS accumulation exerts anti-tumorigenic effects through oxidative stress-mediated cellular damage and activation of tumor cell death pathways [41]. Consequently, advancing our understanding of the regulatory functions and underlying mechanisms of ROS in tumorigenesis is essential for effective cancer prevention and therapy.

#### ROS-Mediated carcinogenesis

When ROS levels surpass cellular antioxidant capacity, they become tumorigenic by inducing DNA lesions and genomic instability in normal cells [42]. Persistent DNA damage disrupts genetic information fidelity, leading to mutations and epigenetic alterations that collectively drive malignant transformation into neoplastic phenotypes [43].

##### ROS promoting tumor cell proliferation

In neoplastic cells, subtoxic concentrations of ROS function as critical secondary messengers that facilitate acquisition and maintenance of oncogenic phenotypes through modulation of key signaling pathways. For example, H_2_O_2_-induced PTEN inactivation hyperactivates the PI3K/AKT/mTOR axis, promoting tumorigenesis [44]. Elevated ROS levels in tumor cells can enhance the MAPK/ERK signalling cascade in a cell-specific manner, thereby stimulating tumour cell proliferation and survival [45]. Additionally, ROS oxidize EGFR at cysteine-797, augmenting its tyrosine phosphorylation and subsequently activating downstream Ras/MAPK and PI3K/AKT pathways to drive tumor growth [46–49]. Collectively, these findings establish ROS as pivotal orchestrators of pro-tumorigenic signaling networks.

##### ROS promoting tumor metastasis

Metastasis—the dissemination of cancer cells from primary tumors to distant organs—represents the leading cause of cancer-related mortality. ROS contribute critically to metastatic processes.

Transforming growth factor-β1 (TGF-β1) enhances tumor cell migration and invasion by upregulating urokinase-type plasminogen activator (uPA) and matrix metalloproteinase-9 (MMP-9) via ROS-dependent signaling [50]. Furthermore, ROS promote metastasis through Rho-GTPase-mediated cytoskeletal remodeling, which facilitates MMP-dependent extracellular matrix degradation and hypoxia-inducible factor (HIF)-driven angiogenesis [51]. These mechanisms underscore the significant regulatory role of ROS in tumor dissemination.

#### Anti- Cancer role of ROS

When ROS accumulation surpasses the cellular threshold in malignant cells, pro-survival effects shift to anti-tumor outcomes. Furthermore, in cancer therapy, cytotoxic ROS levels potentiate the genotoxicity of chemotherapeutic agents, synergistically enhancing treatment efficacy and overcoming drug resistance.

##### ROS and apotosis

ROS act as potent mediators of programmed cell death by concurrently activating multiple apoptotic pathways, including the intrinsic mitochondrial, extrinsic death receptor, and endoplasmic reticulum (ER) stress pathways [52]. The mitochondrial pathway is particularly critical. ROS initiate the intrinsic apoptotic cascade by modulating mitochondrial permeability via interactions with Bcl-2 family proteins (e.g., pro-apoptotic Bax and Bak) [53]. Subsequent mitochondrial permeability transition pore (MPTP) activation causes membrane potential collapse and cytochrome c release, triggering apoptosome formation and caspase activation. The extrinsic pathway is initiated through death ligand-receptor binding (e.g., FasL/Fas and TNF/TNFR), activating downstream apoptotic executers [54, 55].

##### ROS and ferroptosis

**Fig. 5:**
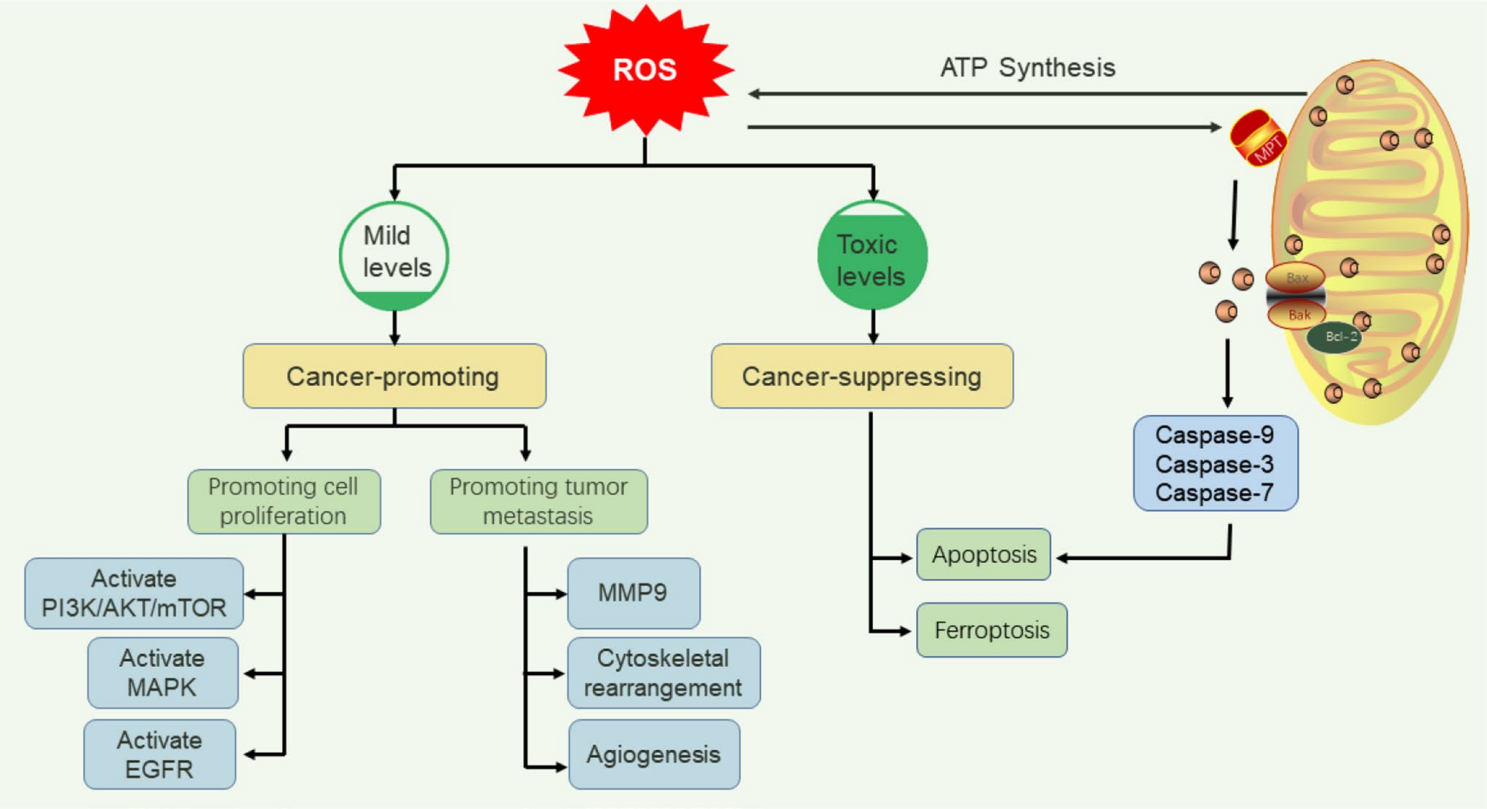
Dual Role of ROS in Tumor Regulation (Created with PowerPoint). ROS exhibit context-dependent effects: moderate levels promote tumor growth (PI3K/AKT, MAPK pathways) and metastasis (TGF-β1/MMP activation, angiogenesis), while excessive ROS induce apoptosis (mitochondrial/death receptor pathways) and ferroptosis (lipid peroxidation via iron/ROS interplay). The threshold of ROS accumulation dictates their pro- or anti-tumor activity, emphasizing the therapeutic potential of ROS modulation

Ferroptosis is an iron-dependent, non-apoptotic programmed cell death modality driven by ROS accumulation [56]. Its execution requires both elevated intracellular iron and ROS-induced peroxidation of polyunsaturated fatty acids (PUFAs) [57]. Disruption of redox homeostasis leads to excessive ROS accumulation, which oxidizes membrane-embedded PUFAs in malignant cells. This lipid peroxidation cascade ultimately induces ferroptotic cell death [58] (Fig. 5).

## The role of ROS and Nrf2 in cellular EMT

### ROS and EMT

#### ROS in EMT-mediated tumor invasion and metastasis

Accumulating evidence identifies ROS as critical secondary messengers that regulate EMT in neoplastic cells. Recent studies reveal that key EMT regulators are redox-sensitive, providing mechanistic insights into ROS-dependent EMT modulation [59, 60]. Through reversible/irreversible oxidation of cysteine residues in redox-sensitive proteins, ROS govern signaling pathways essential for extracellular matrix (ECM) remodeling, cytoskeletal reorganization, cell junction dissolution, and motility enhancement [61]. Pharmacological inhibition of ROS (e.g., via N-acetylcysteine, NAC) effectively suppresses ROS-driven EMT progression [62].

#### ROS in EMT associated cancer stem cell properties

EMT confers cancer stem cell (CSC)-like traits that drive metastasis and therapy resistance. ROS critically regulate CSC property acquisition [63], with ROS-induced EMT generating stem-like phenotypes in specific cancer subtypes [64]. The ROS-antioxidant balance governs CSC plasticity [65]: quiescent CSCs maintain low ROS levels and high glutathione (GSH) for therapy resistance, whereas proliferative CSCs elevate ROS to activate Nrf2-mediated survival pathways. The flexibility of CSCs in transitioning between quiescent and proliferative states provides a critical mechanism for mesenchymal - epithelial transition (MET)and EMT during metastatic progression. Consequently, targeting EMT by modulating intracellular redox homeostasis represents a promising anticancer strategy.

### Nrf2-Mediated EMT regulation via redox signaling

Tumorigenesis requires substantial energy, primarily derived from glucose metabolism with minor contributions from glutamine and fatty acid oxidation [46]. During mitochondrial ATP synthesis in malignant cells, elevated ROS production creates a pro-oxidant microenvironment distinct from normal cells. Tumor progression in this high-ROS milieu involves crosstalk between energy metabolism and oxidative stress. Hypoxic conditions exacerbate ROS accumulation, activating hypoxia-inducible factor (HIF) signaling and subsequent Snail pathway induction [66]. ROS enhance migration, reduce cell adhesion, remodel cytoskeleton, and upregulate mesenchymal markers through HIF-1, p38 MAPK, NF-κB, and PI3K/Akt pathways [67]. Antioxidants (e.g., NAC) can suppress EMT by attenuating ROS generation.

#### Nrf2-Mediated EMT Inhibition

As a master antioxidant regulator, Nrf2 modulates intracellular ROS levels. Given Nrf2’s association with ROS and ROS-dependent EMT regulation, functional crosstalk exists between Nrf2 and EMT. Nrf2 activation inhibits EMT in pulmonary fibrosis by suppressing Numb and Snail1 expression. Mechanistically, Nrf2 transcriptionally activates antioxidant enzymes (glutathione-S-transferase, glutathione peroxidase, catalase) that scavenge ROS and prevent oxidative damage, thereby limiting EMT initiation [68, 69]. Nrf2 pathway dysregulation in tumors enables ROS accumulation, enhancing cellular plasticity and motility [70].

#### Nrf2-Mediated EMT promotion

Paradoxically, Nrf2 activation can promote metastasis by inducing EMT. The ROS/Nrf2/Notch axis upregulates Snail to drive hepatocellular carcinoma invasion [71]. ROS-induced PRDX5 hypomethylation activates Nrf2 signaling, facilitating lung cancer EMT [72]. Nrf2 overexpression enhances breast cancer migration [73], and promotes invasiveness via PI3K/AKT pathway activation [74], and mesenchymal markers modulation (e.g., MMP9) [75]. Collectively, these findings demonstrate Nrf2’s bidirectional EMT regulation.

In conclusion, Nrf2 plays a dual role in EMT regulation through antioxidant and pro-survival signaling. In fibrotic and degenerative diseases, Nrf2 directly antagonizes EMT by suppressing Snail, α-SMA, etc. Below is a comparative table summarizing studies on Nrf2’s dual roles in promoting and inhibiting EMT, based on literature. The table systematically compares model systems, intervention methods, phenotypic indicators, and references (Table 2).

**Table 2:** Pro- VS Anti-EMT effect of Nrf2​

Pro- VS Anti-EMT	Model System	Intervention Method	Phenotypic Indicators
Anti-EMT	Bleomycin-induced pulmonary fibrosis mouse model	Nrf2 knockout (Nrf2−/−)	Snail↑; E-cadherin↓, α-SMA↑; Increased collagen deposition [69]
Anti-EMT	TGF-β1-induced alveolar epithelial cells (RLE-6TN)	Nrf2 activator (SFN) pretreatment	Numb↓; E-cadherin↑, α-SMA↓; HO-1/NQO1 upregulation [68]
Anti-EMT	LPS-induced bovine endometrial epithelial cells (BEECs)	Phellodendron alkaloid (Berberine) activates Nrf2	ROS↓; E-cadherin↑, Vimentin↓ [76]
Anti-EMT	Lung cancer cells (A549, H460)	Hypoxia-induced activation of NRF2/miR-27a axis	Enhanced migration/invasion; E-cadherin↓, N-cadherin↑; BUB1 upregulation [77]
Pro-EMT	Hepatocellular carcinoma (HCC)	MCUR1 overexpression activates ROS/Nrf2/Notch	Snail↑; E-cadherin↓, Vimentin↑; Enhanced cell migration and metastasis [71]
Pro-EMT	Macrophage-cancer cell co-culture model	Cancer cell-derived lactate activates macrophage Nrf2	VEGF secretion↑; Mesenchymal-like cancer cell morphology (E-cadherin↓, N-cadherin↑) [4]
Pro-EMT	Non-small cell lung cancer cells	Nrf2 inhibitor (Brusatol) treatment	RhoA-ROCK1 pathway inhibition; Reduced cell migration [78]

## Tissue-Specific Nrf2-ROS-EMT dynamics in tumor microenvironments

Nrf2 and ROS exhibit reciprocal regulation during EMT: Nrf2 mitigates ROS-induced damage via antioxidant responses, while ROS activate Nrf2 to regulate EMT initiation. Tumor-type-specific variations in ROS generation and Nrf2 activation manifest through divergent ROS sources, regulatory mechanisms, and functional outcomes in tumor progression.

### Heterogeneity in ROS generation pathways

ROS are primarily generated via mitochondrial metabolism, NADPH oxidases (e.g., NOX4), and heme oxygenases. For example, in ​​lung cancer​​, ROS are produced through the mitochondrial respiratory chain (e.g., Complex I and III) [79], whereas in ​​hepatocellular carcinoma (HCC)​​, ROS may arise via iron-dependent pathways [80]. The accumulation levels and regulatory mechanisms of ROS also vary across tumor types. Breast cancer employs the STEAP1-Nrf2 axis for ROS modulation, contrasting with Nrf2-Keap1 pathway regulation in prostate cancer [80].

### Heterogeneity in Nrf2 activation mechanisms

Under physiological conditions, Keap1 sequesters Nrf2 in the cytoplasm. ROS oxidize Keap1 cysteine residues, triggering Nrf2 nuclear translocation and ARE-driven gene expression. In specific tumors, Nrf2 undergoes constitutive activation via mutations or gene amplification. Lung and head/neck cancers exhibit mutation-stabilized Nrf2 enhancing ROS tolerance [81], while breast cancer utilizes Ras/MAPK-dependent Nrf2 activation [82]. ROS-dependent Nrf2 regulation further varies across microenvironments: APE1 mediates ROS-dependent Nrf2 activation in Barrett’s esophageal adenocarcinoma [83], whereas autophagy-related proteins (e.g., SQSTM1/p62) regulate this axis in HCC [84].

### Stage- and Tissue-Dependent roles of Nrf2 in tumor progression

During early tumorigenesis, moderate Nrf2 activation inbibits tumor initiation and cancer metastasis by eliminating carcinogens, ROS and other DNA-damaging agents [82]. Nrf2 regulated cellular defense delays hepatocarcinogenesis in tyrosinemia type 1 (HT1) [85]. In advanced tumors (non-small cell lung carcinoma, endometrial carcinoma and ovarian carcinom), sustained Nrf2 activation enhances ROS tolerance, promoting tumor progression and chemotherapy resistance [86].

### Metabolic Reprogramming by Nrf2

Nrf2 activation not only regulates antioxidant genes but also adapts cells to high ROS environments by modulating glutathione metabolism, glycolysis, and the pentose phosphate pathway (PPP). In triple-negative breast cancer (TNBC), Nrf2 governs glutathione synthesis via PPP flux control [87].

### Integrative analysis of Nrf2-ROS-EMT heterogeneity

Differences in ROS generation pathways and Nrf2 activation modes reflect tissue-specific metabolic demands and microenvironmental variations. ​​Table 3​​ systematically compares the heterogeneity of Nrf2-ROS-EMT regulatory dynamics across tumor types, highlighting tissue-specific adaptations and microenvironmental dependencies.

**Table 3:** Heterogeneity in Nrf2-ROS-EMT regulatory dynamics​

Specific Tumor Type	ROS Source	Nrf2 Role	Tissue-Specific Variation
HCC	Mitochondrial ROS (mROS)	Promotes Notch1 activation, enhancing EMT and metastasis	MCUR1 is uniquely overexpressed in HCC, dependent on mitochondrial calcium signaling [71]
NSCLC	NOX4, mitochondrial ROS	Hypoxia inhibits miR-27a, activating BUB1 → EMT; high ROS activates antioxidant genes	NOX4 dominates ROS generation in NSCLC; miR-27a/BUB1 axis is unique [77]
Breast Cancer	NOX4, mitochondrial ROS	STEAP1 loss reduces Nrf2 activity → weakens antioxidant capacity → enhances EMT	STEAP1 has dual roles in breast cancer (tumor suppression vs. EMT promotion) [80]
Osteosarcoma	Mitochondrial ROS, NADPH oxidase	TRIM22 loss destabilizes Nrf2 → ROS accumulation → autophagy and apoptosis→ EMT suppression	TRIM22 is uniquely downregulated in osteosarcoma, regulating Warburg effect via Nrf2 [88]
MDS	Mitochondrial ROS (iron overload)	Iron chelation therapy (ICT) restores Nrf2 activity → reduces oxidative damage→Atypical EMT	Iron overload uniquely disables NRF2 in MDS [80]
Retinopathy (non-cancer)	Mitochondrial ROS, exogenous oxidants	Nrf2 activation inhibits AKT/GSK-3β → restores E-cadherin	Isorhamnetin uniquely inhibits RPE cell EMT via Nrf2 [89]

Abbreviations:​​ HCC (Hepatocellular Carcinoma); NSCLC (Non-Small Cell Lung Cancer); MDS (Myelodysplastic Syndrome)

## Clinical translational applications of Nrf2/ROS modulators

Nrf2 activators and inhibitors have demonstrated significant potential in clinical trials across multiple disease areas (e.g., neurodegenerative diseases, chronic kidney disease, cancer), though their specific indications and safety profiles require further investigation and validation. Meanwhile, ROS inducers and scavengers are primarily in Phase I and II trials, targeting various cancers and other diseases. Table 4 summarizes the developmental progress and translational strategies of Nrf2/ROS-targeting drugs.

**Table 4:** Nrf2/ROS-Targeted Drug Development Landscape

Drug/Compound Name	Target Action	Clinical Trial Phase	Key Findings
Bardoxolone Methyl	Nrf2 Activator	III	Significantly reduces oxidative stress markers in CKD patients and improves renal function; shows anti-fibrotic potential in IPF.
ML385	Nrf2 Inhibitor (DNA-binding domain blocker)	Preclinical/I	Selectively inhibits Nrf2 activity in KEAP1-mutant NSCLC, enhancing platinum drug sensitivity [74, 90]; suppresses proliferation and induces cell cycle arrest in HNSCC [91].
Bitopertin	Nrf2 Activator (indirect)	II	Inhibits osteoclast differentiation via iron-ornithine axis, improving bone loss in osteoporosis models[92].
Dimethyl Fumarate (DMF)	Nrf2 Activator	Approved	Approved for multiple sclerosis and psoriasis; demonstrates anti-tumor activity in cutaneous T-cell lymphoma [93, 94].
Elesclomol (STA-4783)	ROS Inducer	II	Induces melanoma apoptosis via mitochondrial oxidative stress; enhances antitumor efficacy with paclitaxel [95].
Sulforaphane	Nrf2 Activator/ROS Modulator	II/III	Reduces PSA levels in prostate cancer; enhances radiotherapy sensitivity in NSCLC [94].

Abbreviations:​​ CKD (Chronic kidney disease); NSCLC (Non-Small Cell Lung Cancer); HNSCC (Head and Neck Squamous Cell Carcinoma); IPF (Idiopathic Pulmonary Fibrosis); PSA (Prostate-Specific Antigen)

## Conclusion

The role of ROS in tumor progression has garnered substantial research interest. ROS critically activate EMT through redox-sensitive transcription factors and promote tumor invasion via pathways including transforming growth factor-β (TGF-β), Wnt, Notch, and Hedgehog. Conversely, the Nrf2-Keap1-ARE pathway suppresses ROS accumulation through antioxidant responses. Consequently, Nrf2-mediated modulation of EMT and cancer cell migration may operate through ROS-dependent mechanisms. However, Nrf2’s impact on tumorigenesis remains context-dependent, influenced by signaling crosstalk, cancer type, and microenvironmental factors. Further investigations are warranted to delineate the underlying mechanisms. Therapeutic targeting of redox regulation to inhibit EMT and metastasis represents a promising anticancer strategy.

## Data Availability

No datasets were generated or analysed during the current study.
